# Mortality and risk assessment for anorexia nervosa in acute-care hospitals: a nationwide administrative database analysis

**DOI:** 10.1186/s12888-020-2433-8

**Published:** 2020-01-13

**Authors:** Shunsuke Edakubo, Kiyohide Fushimi

**Affiliations:** Department of Health Policy and Informatics, Tokyo Medical and Dental University Graduate School, S1560/S1568 M&D Tower 1-5-45 Yushima, Bunkyo-ku, Tokyo, 113-8519 Japan

**Keywords:** Anorexia nervosa, Mortality, Risk factor, Sex difference

## Abstract

**Background:**

Anorexia nervosa (AN) is a common eating disorder with the highest mortality rate of all psychiatric diseases. However, few studies have examined inpatient characteristics and treatment for AN. This study aimed to characterise the association between mortality and risk factors in patients with AN in acute-care hospitals.

**Methods:**

We conducted a nationwide, retrospective analysis of the Japanese Diagnosis and Procedure Combination inpatient database. Data extraction occurred from April 2010 to March 2016. We estimated in–hospital mortality and identified independent risk factors, using multivariate logistic regression analysis to examine patient characteristics and physical and psychological comorbidities.

**Results:**

We identified 6937 patients with AN aged ≥12 years in 885 acute-care hospitals. Of these, 361 (5.2%) were male. Male and female participants’ median ages at first admission were 34 (17–65) and 28 (17–41) years, respectively. In total, 195 in-hospital patient deaths, including 22 (6.1%) men and 173 (2.6%) women, it was observed that the unadjusted odds ratio of mortality for male patients was more than twice that for female patients (OR: 2.40, 95% CI: 1.45–3.81). Multivariate logistic regression analysis demonstrated an adjusted odds ratio of 2.19 (95% CI: 1.29–3.73). Age at first hospital admission, percentage of ideal body weight, comorbidities, and hypotension were significantly associated with increased mortality risk, but the frequency of hospitalization, bradycardia, and other psychiatric disorders were not. Treatment in a university hospital was associated with lower mortality risk (odds ratio: 0.45, 95% CI: 0.30–0.67).

**Conclusion:**

The results highlighted sex differences in mortality rates. Potential risk factors could contribute to improved treatment and outcomes. These retrospective findings indicate a need for further longitudinal examination of these patients.

## Background

Anorexia nervosa (AN) is a common eating disorder. It is observed mainly in women aged 10–20 years and is associated with high levels of psychological stress or anxiety related to the pursuit of thinness or fear of fatness [1]. These stress are anxiety are specific to this age group. It is mainly present in socio–economically developed countries and in these countries, the prevalence of AN has increased significantly because of socio-cultural traditions involving the admiration of fashion models and thinner women [2].

In Japan, initial outpatient treatment of stable AN often involves primary care physicians or psychiatrists, and includes interventions such as monitoring weight, nutritional rehabilitation, education for patients and family, or cognitive behavioural therapy. Once patients fail to respond to these initial interventions and demonstrate physical or psychiatric instability, hospitalisation is recommended. When their physical conditions are severe, initial treatment is often provided by internal medicine or critical care physicians who are accustomed to using hemodynamic agents.

AN exhibits various physical and psychiatric complications, depending on severity, including medical problems such as hypotension, bradycardia, and arrythmia due to electrolyte abnormality and infection due to low white blood cell count [3]. A common clinical choice in nutritional rehabilitation could be enteral nutrition with a feeding tube; however, patients may experience various symptoms ranging from abdominal pain or discomfort to refeeding syndrome [3, 4]. Therefore, these complexities of nutritional management result in time and cost consumption in the remission of AN patients’ weight [5, 6].

The complexities of management of issues other than nutrition also worsen the prognoses of AN patients. Of all psychiatric disorders, it is observed that AN is associated with the highest mortality rate, which is five times that observed in the general population according to age and sex [7, 8].

The recovery rates ranged from 0 to 92% in a previous review [9]. One of the reasons for this wide discrepancy could be the variety of definitions of recovery, and another could be the power of the evidence in the guidelines for AN [10–14], which were established based on a limited number of patients because of the accumulation of rare diseases. Consequently, a limited number of epidemiological studies have been conducted; in particular, no studies have examined risk according to patients’ background characteristics such as sex, age, and expressions of clinical symptoms.

The purpose of this study was to explore patients’ demographic characteristics and examine what characteristics were risks for AN mortality, using a large Japanese administrative database.

## Methods

### Study design

We conducted a nationwide, retrospective cohort study using a Japanese administrative inpatient database known as the Diagnosis Procedure Combination (DPC) database, which includes claims and discharge data from more than 1000 participating hospitals and holds information from more than 90% of all tertiary-care emergency hospitals in Japan. The database includes the following patient information: age; sex; primary diagnosis; comorbidities at admission; complications after admission classified according to International Classification of Diseases, 10th revision (ICD-10) codes; medical procedures coded with original Japanese codes; daily records of drug administration and devices used; duration of stay; and discharge status. Dates of hospital admission, surgical and nonsurgical procedures, drugs administered, and discharge are recorded using a uniform data submission format.

Although the DPC database lacks information regarding physiological and laboratory measures (e.g. blood pressure and the results of blood tests), it has been validated for many other outcomes, types of exposure, and comorbidities.

### Patient selection

Data from April 2010 to March 2016 were used for this study. To identify patients with AN, we extracted patient data from the database according to the following procedure. We extracted data for patients diagnosed with an eating disorder (ICD-10 codes: F500, F501, F502, F503, F504, and F505) as the main disease, the reason for admission, or a comorbid disorder. We then selected patients whose body weight during hospitalization was below 75% of their ideal weight. As some patients’ weight was more than 75% of their ideal weight because of binge eating, we included patients who had been hospitalised with weight of less than 75% of their ideal weight at least once. We excluded patients who: 1) were younger than 11 years of age; 2) were discharged or died within 2 days of admission, to avoid immortal time bias [15]; 3) were below 1 m in height, which is less than − 3 standard deviations (*SD*s) of the mean height for 11-year-old children [16]; 4) had missing data for weight; or 5) had been diagnosed with digestive carcinoma as they could not, rather than would not, eat.

### Study variables and outcomes

Variables included age, sex, thinness index (defined as the ratio of actual weight to their ideal weight. We calculated ideal weight according to Kato et al.’s methods [16] if they were younger than 18 years or body mass index (BMI) of 22 if they were 18 years or older. As part of school health, body height and weight of the students are recorded officially [17]. According to these data, ideal adolescent weight by age and gender were different from adults: BMI 22 is not an ideal indicator especially for the younger generation in Japan), updated Charlson Comorbidity Index calculated via Quan et al.’s algorithms [18]: predicts 10-year survival in patients with multiple comorbidities, hospital type (i.e. university or non-university), frequency of hospitalisation, use of catecholamine within the first 48 h of admission, commonly prescribed for hypotension, use of atropine within the first 48 h of admission, commonly prescribed for bradycardia, other psychological disorders, and binge eating and purging. The main outcome of the study was all-cause in-hospital mortality.

### Statistical analysis

Descriptive statistics are presented as means and *SD*s or medians with interquartile ranges (IQRs) for continuous variables and frequencies with percentages for categorical variables. Comparisons of continuous variables were performed using the Wilcoxon rank sum test, because the distribution of the continuous variables was far from the normal distribution. The chi-square test was used to compare categorical variables. We calculated the all-cause in-hospital mortality rate with an exact 95% CI. Multivariate logistic regression models were created to identify potential risk factors for in-hospital mortality.

### Subgroup analysis

Two subgroup analyses were performed. The first subgroup analysis was restricted to patients below 60 years to make the diagnosis credible, as a previous study [19]. The second subgroup analysis was conducted on patients who survived. As a cycle of remission and exacerbation is common in eating disorders, prognoses are likely to worsen. Therefore, some patients are admitted to hospital repeatedly. However, the effects of baseline characteristics and treatment on repeated admissions remain unclear. Consequently, in the second subgroup analysis, we investigated the factors that influenced readmission.

The factors examined included patient characteristics, hospital type, and treatment. We selected treatment factors according to the following variables: major surgery such as craniotomy, open heart surgery, thoracotomy, laparotomy, general anaesthesia, blood transfusions, administration of anti–arrhythmia agents, intravenous hyperalimentation, and psychotherapy. These factors were also analysed via a multivariate logistic regression model.

### Sensitivity analysis

To ensure the robustness of the analysis, we conducted sensitivity analysis including the treatment effects used in the subgroup and original analyses.

All analyses were performed using R software version 3.4.3 (R Foundation for Statistical Computing, Vienna, Austria). The analyses were two-tailed, and *p* values of <.05 were considered statistically significant.

## Results

Over seven years, 6937 patients from 885 hospitals met the inclusion criteria. They represented 11066 hospitalisation occurrences; 9165 (82.8%) occurrences were for psychiatric reasons and 1901 (17.2%) were for medical/surgical reasons. Of these patients, 361 (5.2%) were men and represented 465 (4.2%) hospitalization occurrences, and 6576 (94.8%) were women and represented 10601 (95.8%) hospitalization occurrences. Patient characteristics are shown in Table 1.

**Table 1:** Patient characteristics and in-hospital deaths

	*n* (%)	Deaths (%)	*p* value
Total	6937	195 (2.8)	
Sex			<.001
Male	361 (5.2)	22 (6.1)	
Female	6576 (94.8)	173 (2.6)	
Age at first hospitalization			0.009
12–15	1373 (19.8)	2 (0.0)	
16–19	942 (13.6)	7 (0.1)	
20–29	1262 (18.2)	18 (0.3)	
30–39	1305 (18.8)	41 (0.6)	
40–49	1060 (15.3)	41 (0.6)	
50–59	485 (7.0)	27 (0.4)	
60–69	196 (2.8)	17 (0.2)	
70–79	148 (2.1)	15 (0.2)	
≥ 80	166 (2.4)	27 (0.4)	
Proportion of actual weight to ideal weight (*SD*)			<.001
	61.5 (9.1)	55.0 (9.4)	
Charlson Comorbidity Index			<.001
0	5913 (85.2)	127 (1.8)	
1	189 (2.7)	13 (0.2)	
2	724 (10.4)	38 (0.5)	
≥ 3	111 (1.6)	17 (0.2)	
Hospital Type			<.001
University	3047 (43.9)	163 (2.3)	
Non-university	3890 (56.1)	32 (0.5)	
Frequency of hospitalization			0.128
1	4940 (71.2)	154 (2.2)	
2	1141 (16.4)	23 (0.3)	
3	401 (5.8)	10 (0.1)	
4	210 (3.0)	2 (0.0)	
5	70 (1.0)	3 (0.0)	
≥ 6	166 (2.4)	3 (0.0)	
Maximum	30	8	
Physical status at admission			
Bradycardia	43 (0.6)	1 (0.0)	0.999
Hypotension	146 (2.1)	34 (0.5)	<.001
Other psychological disorder			
Anxiety disorder	678 (9.8)	7 (0.1)	0.002
Mood disorder	898 (12.9)	20 (0.3)	0.329
Schizophrenia	533 (7.7)	12 (0.2)	0.496
Binge eating and purging	745 (10.7)	10 (0.1)	0.009

Participants’ median ages at first hospitalization were 34 (IQR: 17–65) for male patients and 28 (IQR: 17–41) for female patients (*p* < .001). The results showed that there were 195 in-hospital deaths (2.8%; 95% CI: 2.4–4.3%), which included 22 men (6.5%; 95% CI: 3.9–9.1%) and 173 women (2.6%; 95% CI: 2.2–3.0%). It was observed that the unadjusted odds ratio of mortality for male patients was more than twice that for female patients (OR: 2.40, 95% CI: 1.45–3.81, *p* < .001).

The percentages of ideal weight were relatively low for deceased patients (61.7% for alive patients, 55.0% for deceased patients).

Table 1 also shows the physical and psychological complications for all patients and cases involving death. For approximately 90% of patients, Charlson Comorbidity Index values were < 2. This indicated that the majority of eligible patients were basically healthy. Hemodynamic instability was rare, and the complication rate for psychological disorders was approximately 10%.

The results of the multivariate logistic regression model are shown in Table 2. The model showed good discrimination (c statistic = 0.935; 95% CI: 0.918–0.952). After adjustment for multiple potential risk factors, male sex showed an OR of 2.19 (95% CI: 1.29–3.73, *p* = .004) for risk of mortality. Among the potential risk factors, age, thinness index, comorbidities, hospital type, and use of catecholamines were significantly associated with mortality; however, frequency of hospitalization, use of atropine, other mental disorders, and binge eating and purging were not. Treatment in a university hospital was associated with a lower OR, relative to those observed for other risk factors, for in-hospital mortality.

**Table 2:** Risk factors for in-hospital deaths: multivariate logistic regression

	OR	95% CI	*p*-value
Sex (ref. female)			
Male	2.19	1.29–3.73	.004
Age at first admission	1.05	1.04–1.06	<.001
Ratio of actual weight to ideal body weight (thinness index)	0.92	0.90–0.94	<.001
Charlson Comorbidity Index	1.27	1.14–1.42	<.001
Hospital type (ref. non-university)			
University	0.45	0.30–0.67	<.001
Frequency of hospitalization	0.92	0.79–1.08	.322
Use of catecholamine	8.65	5.45–13.7	<.001
Use of atropine	0.27	0.03–2.12	.213
Other psychological disorders			
Anxiety disorder	0.47	0.22–1.04	.063
Mood disorder	0.80	0.49–1.31	.376
Schizophrenia	0.99	0.53–1.86	.976
Binge eating and purging	1.47	0.71–3.02	.296

Table 3 shows the results of a subgroup analysis using age-restricted patients. The results of the age-restricted analysis were similar to those observed in the main analysis (OR: 2.40, 95% CI: 1.00–5.73 for males compared to females).

**Table 3:** Risk factors for patients aged under 60 years in 6454 patients: multivariate logistic regression

	OR	95% CI	*p*-value
Sex (ref. female)			
Male	2.40	1.00–5.73	.049
Age at first admission	1.04	1.03–1.06	<.001
Ratio of actual weight to ideal body weight (thinness index)	0.91	0.89–0.93	<.001
Charlson Comorbidity Index	1.28	1.11–1.49	<.001
Hospital type (ref. non-university)			
University	0.45	0.30–0.69	<.001
Frequency of hospitalization	0.93	0.79–1.10	.399
Use of catecholamine	9.82	6.00–16.10	<.001
Use of atropine	0.27	0.03–2.19	.221
Other psychological disorders			
Anxiety disorder	0.54	0.23–1.27	.157
Mood disorder	0.83	0.45–1.52	.550
Schizophrenia	0.72	0.34–1.54	.397
Binge eating and purging	1.69	0.78–3.65	.180

The results of the subgroup analysis on the risk for re-admission is shown in Table 4. In contrast to the main analysis, the OR for men was small relative to that observed for women (OR: 0.60, 95% CI: 0.43–0.84). Most factors were related to the risk of re–admission.

**Table 4:** Risk factors related to re-admission: multivariate logistic regression

	OR	95% CI	*p*-value
Sex (ref. female)			
Male	0.60	0.43–0.84	.003
Age at first admission	0.99	0.99–0.99	.008
Ratio of actual weight to ideal body weight (thinness index)	0.99	0.98–0.99	<.001
Charlson Comorbidity Index	1.28	1.12–1.38	<.001
Hospital type (ref. non-university)			
University	1.47	1.30–1.67	<.001
Use of catecholamine	1.90	1.19–3.03	.007
Use of atropine	0.67	0.29–1.54	.341
Other psychological disorders			
Anxiety disorder	1.94	1.60–2.36	<.001
Mood disorder	1.76	1.47–2.09	<.001
Schizophrenia	2.58	2.09–3.19	<.001
Binge eating and purging	32.60	19.7–54.10	<.001
Psychotherapy	2.56	2.01–3.26	<.001
Intravenous hyperalimentation	1.99	1.75–2.26	<.001
Major surgery	1.87	0.85–4.15	.123
General anaesthesia	2.12	1.28–3.52	.004
Blood transfusion	0.70	0.45–1.10	.122
Use of anti-arrhythmia agents	1.98	0.94–4.17	.072

The results of the sensitivity analysis, including treatment effects, are shown in Table 5. Of the treatment factors, major surgery was not associated with higher OR (OR: 0.98, 95%CI: 0.13–7.52). After adjustment for treatment effects, mortality for men remained approximately twice that observed for women (OR: 1.94, 95% CI: 1.12–3.38).

**Table 5:** Risk factors for in-hospital deaths: multivariate logistic regression

	OR	95% CI	*p*-value
Sex (ref. female)			
Male	1.94	1.12–3.38	.019
Age at first admission	1.04	1.03–1.05	<.001
Proportion of actual weight to ideal body weight (thinness index)	0.93	0.91–0.95	<.001
Charlson Comorbidity Index	1.22	1.09–1.37	<.001
Hospital type (ref. non-university)			
Academic	0.48	0.31–0.75	.001
Frequency of hospitalization	0.93	0.79–1.09	.373
Use of catecholamine	3.76	2.21–6.39	<.001
Use of atropine	0.07	0.06–0.91	.042
Other psychological disorders			
Anxiety disorder	0.59	0.26–1.33	.203
Mood disorder	0.80	0.48–1.35	.406
Schizophrenia	1.03	0.52–2.03	.938
Binge eating and purging	0.20	0.09–0.45	<.001
Psychotherapy	0.38	0.27–0.53	<.001
Intravenous hyperalimentation	2.25	1.54–3.28	<.001
Major surgery	0.98	0.13–7.52	.985
General anaesthesia	0.11	0.002–0.46	.003
Blood transfusion	9.81	6.13–15.7	<.001
Use of anti-arrhythmia agents	5.23	2.25–12.2	<.001

## Discussion

To our knowledge, this was the first study conducted using a large-scale database to demonstrate sex differences in risk factors in patients with AN. These data showed that the in-hospital mortality rate for this patient group was approximately 3%, and the mortality rate for male patients was more than twice that for female patients. This increase was not explained by differences in patients’ baseline characteristics, clinical expression, or other mental disorders.

Only one previous study conducted in a single centre showed that the 3-year mortality rate for male AN patients was higher than for female patients [20]. The authors hypothesised that men received less social support after discharge than women. The current study showed that men’s age at first hospitalization was higher relative to that of women. One possible explanation for this finding could be that delayed intervention or insufficient recognition of the severity by both patients and medical staff contributed to the worsening of outcomes.

In the subgroup analysis, we demonstrated that men were less likely to be re-admitted relative to women. This result could be explained by sex differences. Male patients could have received inadequate interventions and been hospitalised only when they were in a more fragile state, relative to female patients, for various reasons. The lack of an association between the frequency of hospitalization and higher mortality rates in the main analysis could also explain this. These findings suggest that early and multiple advanced medical interventions could be favourable. AN is relatively rare and clinically atypical in male patients; therefore, it requires careful treatment.

We also demonstrated for the first time that treatment at a university hospital exerted a favourable effect on mortality. The management of AN patients is complicated by not only psychotherapy but also supportive care. For example, titration of drug administration is important, as abnormal distribution volumes could cause pharmacodynamics to differ from those observed in healthy individuals, and refeeding syndrome during enteral nutrition could also be a critical problem [21–23]. The newest ESPEN guidelines support multimodal nutrition for patients at high risk of preoperative conditioning [24]. From these perspectives, involving several experts in treatment is reasonable, and the lower OR observed for university hospitals, relative to those observed for other hospitals, could have resulted from the involvement of many experts and departments in patient care, which is emphasised by several guidelines and reports [10–14, 25]. The findings of the current study support the notion that multi-professional interventions contribute to better outcomes.

The sensitivity analysis showed several treatment risks. For example, critical hypotension, arrhythmia, and blood transfusions were associated with higher mortality, but major operations and general anaesthesia were not. According to the “obesity paradox,” underweight patients are at increased risk of mortality in general surgical procedures relative to patients of a healthy weight [26, 27]. Improvement of the management of these conditions via short-acting drugs or advanced monitoring devices could contribute to better outcomes. The results indicated that when patients require surgical intervention, practitioners should not hesitate to provide this, as there might not be time to hesitate.

The study was subject to several limitations. For example, as the DPC database is based on diagnoses recorded by attending physicians, these diagnoses could have been less valid relative to those in prospective studies. Due to the limitation in that we could not access behavioural criteria (i.e. pursuit of thinness or fear of fat) or associated symptoms, our samples might include individuals with avoidant-restrictive food intake disorder as classified in DSM-V or other low weight conditions.

In addition, the DPC database does not include clinical or physiological information such as that regarding hypotension or arrhythmia. Although we substituted the use of medications for this information, we could not adjust for these factors using actual blood pressure values or types of arrhythmia.

Moreover, we could not identify the history or duration of AN or the time of onset. Therefore, we could not identify medical or psychiatric treatment before hospitalization. We examined the data for a long period (7 years); however, because AN is a disease that is sometimes treated over the life span [28], this could have been insufficient.

Further, because of the database design, our assessment of hospital readmission was limited to individuals admitted to the same hospital. Therefore, we could identify only individuals using the same index if they had been readmitted to the same hospital. If patients were admitted to different hospitals, they were regarded as different patients with different index values. Therefore, we could have duplicated data for some patients, and the actual number of patients could have been lower.

The database design was also a study limitation, as when patients received home–based care or were admitted to psychiatric hospitals, their data were not included in the DPC database, and their mortality rate was unknown. As psychiatric hospitals are relatively scarce (approximately one-seventh of traditional hospitals in Japan [29], this factor could have exerted a weak impact on overall mortality.

## Conclusion

This study showed that the risk of mortality in male AN patients was more than twice that of female AN patients. Age at first admission, the proportion of actual weight to ideal body weight, comorbidities, and hypotension were potential risk factors for mortality. However, the receipt of treatment at university hospitals exerted a favourable effect. Major surgery and general anaesthesia may not worsen the outcomes. Recognition of and preparation for the risk factors identified in this study could contribute to improved outcomes. Further longitudinal examination of AN patients is required.

## Data Availability

The datasets used and/or analysed during this study are available from the corresponding author on reasonable request.

## Abbreviations

AN: Anorexia nervosa
BMI: Body mass index
CI: Confidence interval
DPC: Diagnosis and procedure combination
ICD-10: International classification of diseases, 10th revision (ICD-10)
IQR: Interquartile range
OR: Odds ratio
*SD*: Standard deviation
